# A missense mutation in *SLC26A3* is associated with human male subfertility and impaired activation of CFTR

**DOI:** 10.1038/s41598-017-14606-3

**Published:** 2017-10-27

**Authors:** Satu Wedenoja, Ahlam Khamaysi, Liana Shimshilashvili, Shireen Anbtawe-Jomaa, Outi Elomaa, Jorma Toppari, Pia Höglund, Kristiina Aittomäki, Christer Holmberg, Outi Hovatta, Juha S. Tapanainen, Ehud Ohana, Juha Kere

**Affiliations:** 1 Obstetrics and Gynecology, University of Helsinki and Helsinki University Hospital, FI-00014 Helsinki, Finland; 2 Department of Clinical Biochemistry and Pharmacology, Faculty of Health Sciences, Ben-Gurion University of the Negev, Beer-Sheva, Israel; 3 Folkhälsan Institute of Genetics, and Molecular Neurology Research Program, University of Helsinki, FI-00014 Helsinki, Finland; 4 Department of Physiology, Institute of Biomedicine, University of Turku, and Department of Pediatrics, Turku University Hospital, FI-20014 Turku, Finland; 5 City of Kauniainen, Health Care Services, FI-02700 Kauniainen, Finland; 6 HUSLAB, Laboratory of Genetics, Helsinki University Hospital, and Genome-Scale Biology research program, University of Helsinki, FI-00029 Helsinki, Finland; 7 Hospital for Children and Adolescents, University of Helsinki and Helsinki University Hospital, FI-00014 Helsinki, Finland; 8 Department of Clinical Science, Karolinska Institutet, SE-17177 Stockholm, Sweden; 9 Obstetrics and Gynecology, University of Oulu and Oulu University Hospital, FI-90220 Oulu, Finland; 10 Department of Biosciences and Nutrition, Karolinska Institutet, SE-14183 Huddinge, Sweden; 11 Department of Medical & Molecular Genetics, King’s College London, London, SE1 9RT England

## Abstract

Chloride absorption and bicarbonate excretion through exchange by the solute carrier family 26 member 3 (SLC26A3) and cystic fibrosis transmembrane conductance regulator (CFTR) are crucial for many tissues including sperm and epithelia of the male reproductive tract. Homozygous *SLC26A3* mutations cause congenital chloride diarrhea with male subfertility, while homozygous *CFTR* mutations cause cystic fibrosis with male infertility. Some homozygous or heterozygous *CFTR* mutations only manifest as male infertility. Accordingly, we studied the influence of *SLC26A3* on idiopathic infertility by sequencing exons of *SLC26A3* in 283 infertile and 211 control men. A heterozygous mutation c.2062 G > C (p.Asp688His) appeared in nine (3.2%) infertile men, and additionally, in two (0.9%) control men, whose samples revealed a sperm motility defect. The p.Asp688His mutation is localized in the CFTR-interacting STAS domain of SLC26A3 and enriched in Finland, showing a significant association with male infertility in comparison with 6,572 Finnish (*P* < 0.05) and over 120,000 global alleles (*P* < 0.0001) (ExAC database). Functional studies showed that while SLC26A3 is a strong activator of CFTR-dependent anion transport, SLC26A3-p.Asp688His mutant retains normal Cl^−^/HCO_3_^−^ exchange activity but suppresses CFTR, despite unaffected domain binding and expression. These results suggest a novel mechanism for human male infertility─impaired anion transport by the coupled SLC26A3 and CFTR.

## Introduction

Approximately 15% of couples face infertility, to which male factor is a major contributor in 30–40% of the cases^1^. While over 2000 genes are involved in human spermatogenesis, genetic testing for male infertility is largely unavailable. In clinical practice, analyses of karyotype, Y-chromosomal microdeletions, and mutations in the *cystic fibrosis transmembrane conductance regulator* (*CFTR)* gene (MIM: 602421) play a central role^2^. In the search for rare single-gene variants for sperm defects, however, the presumed low effect of single polymorphisms, the complexity of the phenotype and pedigree studies, and the lack of viable animal models for human spermatogenesis make studies challenging^3^.

A rare autosomal recessive disorder with male subfertility is congenital chloride diarrhea (CLD [MIM: 214700]). It is caused by homozygous or compound heterozygous mutations in the *solute carrier family 26 member 3* (*SLC26A3* alias *DRA)* gene (MIM: 126650) which disrupt the apical epithelial Cl^−^ absorption and HCO_3_^−^ secretion not only in the intestinal epithelia but also at multiple sites of the male reproductive tract^4–7^. This impairment results in oligoasthenoteratozoospermia (OAT), high seminal plasma chloride with a low pH, and a tendency to form spermatoceles^8^. Accordingly, *Slc26a3* knock-out mice exhibit reduced fertility with a 50% reduction in the number of pups compared with wild-type mice^9^. Both CFTR and SLC26A3 are endogenously expressed in developing and mature sperm and in the luminal membrane of the male reproductive tract epithelia^10–13^. They interact and reciprocally regulate each other through binding of the R domain of CFTR and the STAS domain of SLC26A3^14,15^. Not only SLC26A3 but also several SLC26 family members bind through their STAS domains to the R domain of CFTR and act as activators of CFTR. This mechanism elevates transepithelial HCO_3_^−^ and fluid secretion and is essential to the CFTR channel function both *in vitro* and *in vivo* ^14–23^. A compromised function of the SLC26 family members is associated with the primary defect of cystic fibrosis (CF [MIM: 219700])—impaired Cl^−^ absorption in conjunction with aberrant HCO_3_^−^ and fluid secretion—through the inability of CFTR mutants to activate the Cl^−^/HCO_3_^−^ exchange mediated by the SLC26 family members, most notably SLC26A3 and/or SLC26A6^14,15,24–27^.

Whereas the impact of *CFTR* defects on male infertility has been extensively studied, the effects of *SLC26A3* variants on male infertility without CLD remain poorly understood. In men with CF, homozygous *CFTR* mutations cause congenital bilateral absence of the vas deferens (CBAVD) and obstructive azoospermia^28^. Notably, *CFTR* variants are also associated with male infertility without CF. Either one or two *CFTR* mutations appear in approximately 80% of the CBAVD cases without CF, and non-CBAVD related male infertility with reduced sperm quality is associated with homozygosity or heterozygosity for less-pathogenic *CFTR* mutations^29–36^. A shortened tract of five thymines (*5 T*) on the splicing region of intron 8 (IVS8) of *CFTR* explains up to 40% of CBAVD cases without CF, and due to its incomplete penetrance, appears also in healthy individuals or in patients with non-classic CF^30,32,37–39^.

The importance of both CFTR and SLC26A3 functions in the physiology of male fertility is supported by their molecular interaction, by the male infertility phenotypes of CF and CLD, and by their role in rodent sperm motility and capacitation^11–13^. These findings prompted us to study whether *SLC26A3* variants are associated with idiopathic male infertility, similar to *CFTR* variants that cause male infertility without CF.

## Results

We observed that, altogether, 25 men with idiopathic infertility carried heterozygous variants in the coding region of the *SLC26A3* gene (GenBank: NM_000111.2), the frequency of heterozygosity being 3.7-fold higher in infertile men than in controls (8.8% vs. 2.4%, respectively; *P* = 0.007) (Table 1). In addition to the CLD-causing mutation c.949_951delGTG (p.V318del)^4^, however, only the missense change c.2062 G > C (p.Asp688His) was predicted deleterious by PolyPhen-2^40^ (Table 1).

**Table 1:** Heterozygous *SLC26A3* variants in infertile and control men.

Exon No.	Sequence variation	Infertile men (n = 283) n (%)	Control men (n = 211) n (%)	*P* value	SNP identity	PolyPhen-2 class (score)
3	c.241 A > G (p.Ile81Val)	1 (0.4)	0		rs116793431	Benign (0.044)
4	c.357 C > A (p.Phe119Leu)	13 (4.6)^a^	3 (1.4)	<0.05	rs73419912	Benign (0.003)
8	c.949_951delGTG (p.Val318del)	3 (1.1)	0		rs121913029	Finnish founder mutation for CLD
18	c.2062 G > C (p.Asp688His)	9 (3.2)^a^	2 (0.9)	<0.05	rs191547831	Probably damaging (0.994)

^a^One subject was a compound heterozygote for p.Phe119Leu/p.Asp688His. The c.921 T > G (p.C307W) variant, a functionally neutral change always preceding the founder mutation c.949_951delGTG (p.Val318del) for CLD^4,5^ was common in both the infertile (8%) and control (14%) group and was excluded from the analysis. *P* values were calculated with one-tailed Chi-square test without Yates’ correction. Only *P* values < 0.05 are shown.

Search from the ExAC database^41^ showed that c.2062 G > C (p.Asp688His) (rs191547831) is a very rare variant, found heterozygous in nine infertile and two control men of this study, and previously in only 106 individuals globally, with no homozygotes. The global minor allele frequency (MAF) is only 0.0009 (106/120,154 alleles): 47/6,572 in Finland (MAF 0.007), 54/66,034 Non-Finnish European (MAF 0.0008), 1/16,314 in South Asia (MAF 6.13 × 10^−5^), and 4/13,006 European-American (MAF 0.0003; NHLBI Grand Opportunity Exome Sequencing Project^42^). Therefore, the variant c.2062 G > C (p.Asp688His) seems to be highly enriched in Finland and shows association with male infertility in comparison with 6,572 Finnish (*P* < 0.05) and over 120,000 global alleles (*P* < 0.0001) (ExAC database)^41^ (Table 2).

**Table 2:** Allele frequencies of the c.2062 G > C (p.Asp688His) variant.

c.2062 G > C, p.Asp688His (rs191547831)	Mutant allele (n)	Normal allele (n)	MAF	*P* value
Infertile men of this study (n = 283)	9	557	0.016	
Control men of this study (n = 211)	2	420	0.005	<0.05
Finnish people (ExAC) (n = 3,286)	47	6,525	0.007	<0.05
European people (ExAC) (n = 33,017)	54	65,980	0.0008	<0.0001
All ExAC men (n = 33,281)	59	66,503	0.0009	<0.0001
All ExAC people (n = 60,077)	106	120,048	0.0009	<0.0001

MAF = minor allele frequency. ExAC = The Exome Aggregation Consortium^41^. *P* values were calculated with one-tailed Chi-square test without Yates’ correction.

Clinical data of the c.2062 G > C (p.Asp688His) heterozygotes (n = 9) among infertile men revealed one azoospermic subject, and others with OAT or severe OAT. In 2 of them, the detected heterozygous *5 T* allele might be an additional contributor to OAT while no other *CFTR* mutations were found. We studied the heterozygous c.2062 G > C (p.Asp688His) allele in 2 controls with unspecified fertility status and observed absence of rapid progressive motility of sperm (WHO class A 0%) in their semen samples while seminal plasma pH, sperm concentrations (15.5, and 64.5 × 10^6^/mL), and morphology (3% and 9%, respectively) were within the normal range. Those three heterozygotes for the Finnish founder mutation for CLD, c.949_951delGTG (p.Val318del) (Table 1), had severe OAT and, in one of the subjects, azoospermia to which an additional contributor may be the heterozygosity of the *CFTR 5 T* allele.

**Figure 1:**
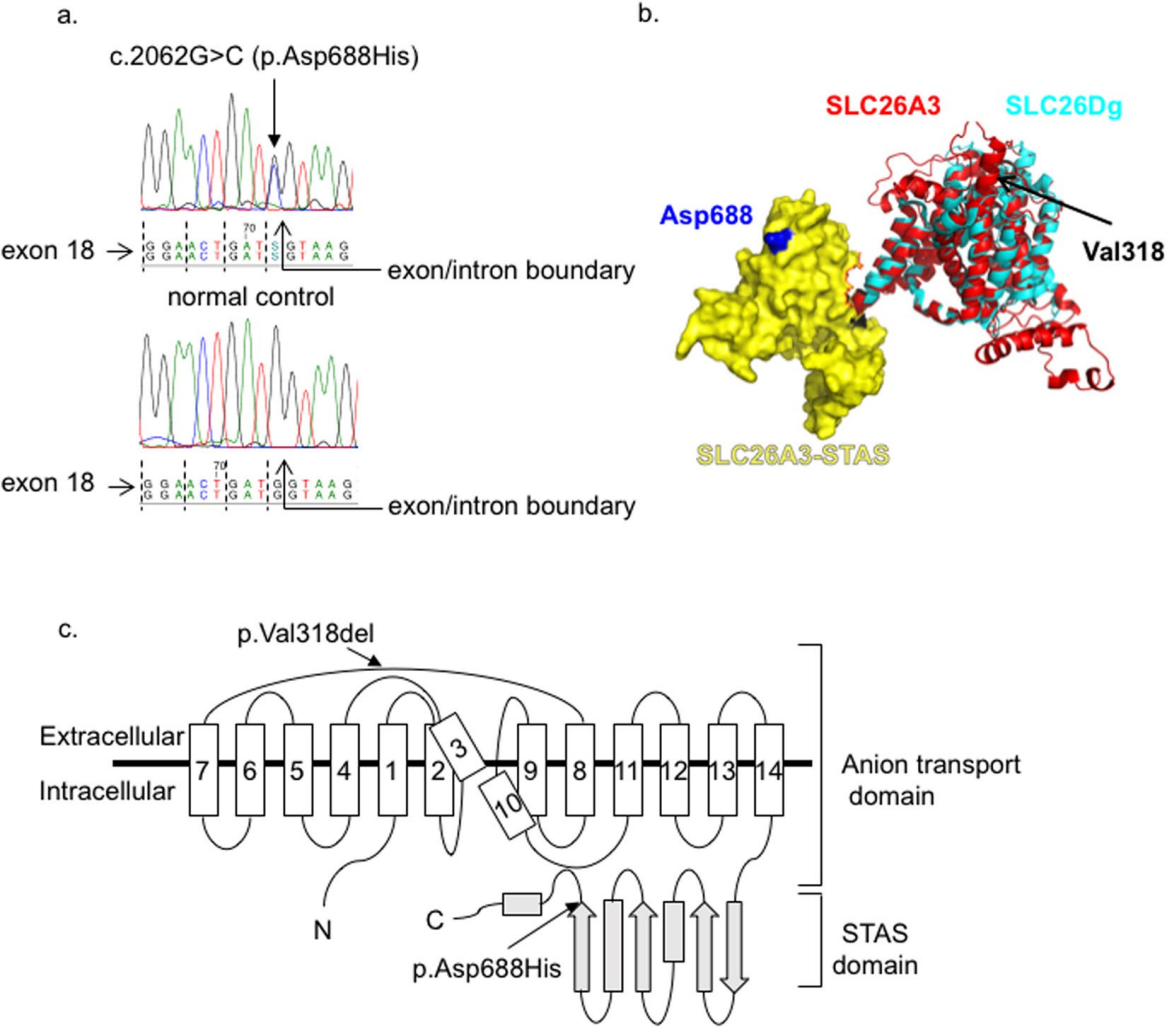
Localization of the c.2062 G > C (p.Asp688His) variant. (**A**) The c.2062 G > C (p.Asp688His) variant is located at the last nucleotide position of the exon 18 of the *SLC26A3* gene. Dashed lines indicate the reading frame of exon 18. (**B**) Putative 3D structure of the human SLC26A3. The transmembrane domain (ribbon, red) and the STAS domain (surface, yellow) representations of the human SLC26A3 (encompassing residues 1–699 of 764) were predicted based on their homology to the SLC26Dg structure (PDB_ID: 5DA0) (ribbon, cyan)^43^ by using Robetta online software (confidence 0.507600)^67^. The final model was generated using PyMol software (Schrödinger, Cambridge, MA). (**C**) The predicted 2D topology of the human SLC26A3 protein. The full-length protein (764 amino acids, AA) comprises 14 putative transmembrane domains—which form the ion transport domain—and the cytoplasmic C-terminal STAS domain. Arrows indicate the approximate positions of the Finnish founder mutation for CLD (c.949_951delGTG, p.Val318del) and for the c.2062 G > C (p.Asp688His) variant^43^. Note: In both the Slc26Dg crystal structure and our putative SLC26A3 model (**B**), the STAS domain orientation in respect to the transmembrane domain (TMD) suggests that the STAS domain is located within the phospholipid bilayer. Hence, this does not represent a native orientation since the STAS domain is expected to be found in the cytoplasmic C-terminal part of the protein.

**Figure 2:**
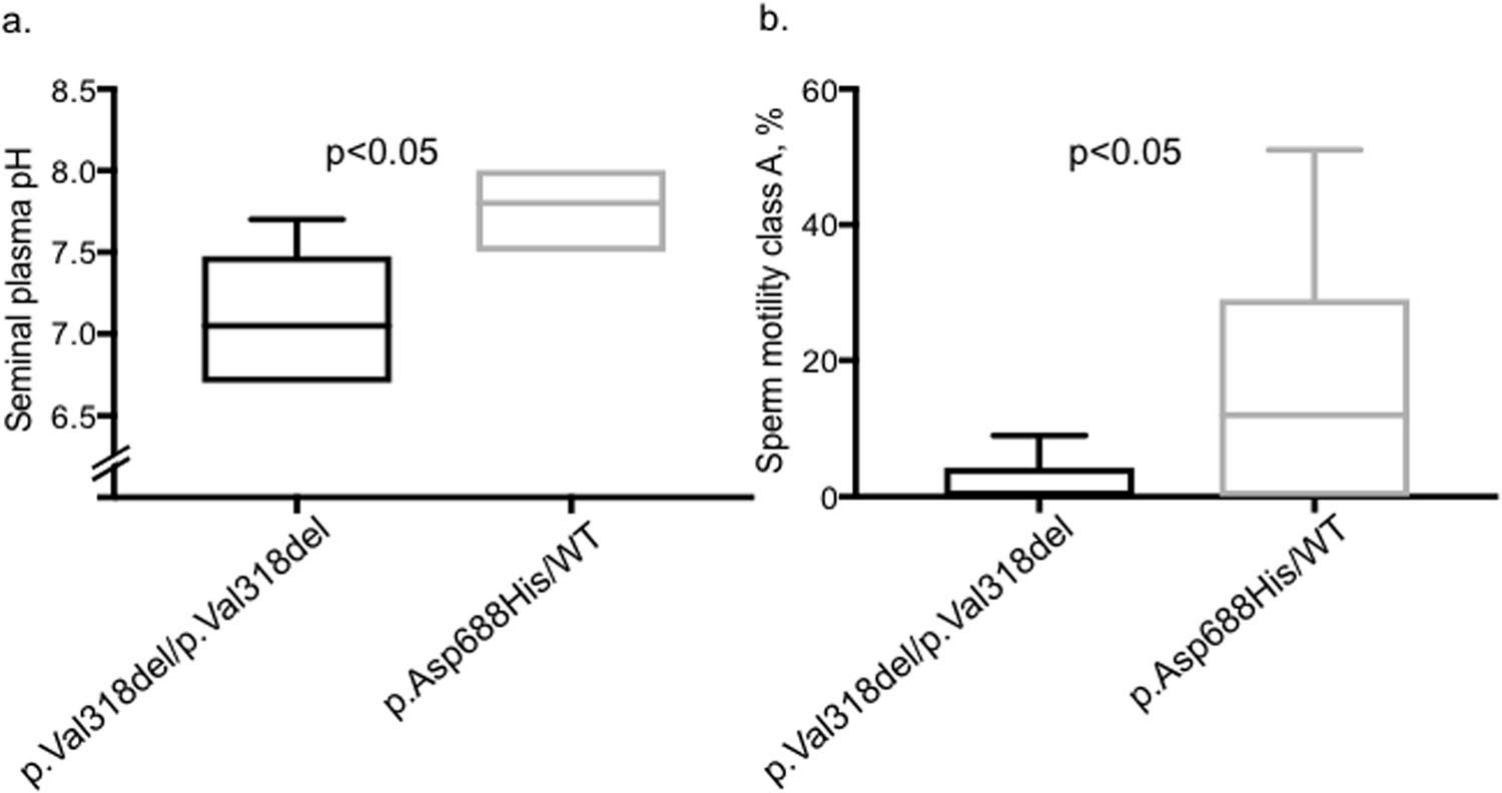
A milder effect of the heterozygous p.Asp688His variant than the homozygous p.Val318del mutation on seminal pH and sperm motility. (**A**) Seminal plasma pH and (**B**) sperm motility class A (%), as found in the semen samples from infertile men (n = 9) carrying the heterozygous variant c.2062 G > C (p.Asp688His) and from men with CLD (n = 8) caused by the homozygous loss-of-function mutation c.949_951delGTG (p.Val318del)^8^. Data are presented from minimum to maximum with the band inside the box indicating the mean value. Two azoospermic subjects, one in each subgroup, were excluded from the motility analysis. The median sperm motility class A in the heterozygotes for the p.Asp688His variant  was 10% and the mean 16%, with 4 of those 8 subjects included in the analysis having the value of ≤2%. WHO normal values are: pH 7.2–7.8 and rapid progressive sperm motility class A > 25%^63^. *P* values were calculated with Student’s *t*-test. WT = wild-type.

The c.2062 G > C missense change resides at the last nucleotide position of the exon 18, and codes for the p.Asp688His change in the intracellular C-terminal STAS domain of SLC26A3, rather than in the transmembrane domain which harbors the ion-transport pathway (Fig. 1). This residue is highly conserved and the histidine replacement is found neither in any animal homologues of *slc26a3* nor in other human *SLC26* family members, giving support to a deleterious role of this sequence variant^40,43^. As SLC26A3 STAS domain directly binds and interacts with the CFTR R domain^15^, we next assessed the functional role of the c.2062 G > C (p.Asp688His) variant  and its potential effect on the activity of CFTR. In addition, we compared the effect of the heterozygous p.Asp688His variant vs. the homozygous p.V318del mutation for CLD^8^ in terms of seminal plasma pH and sperm motility, and found normal pH in association with a milder defect in sperm motility in the 2062 G > C (p.Asp688His) heterozygotes (Fig. 2).

**Figure 3:**
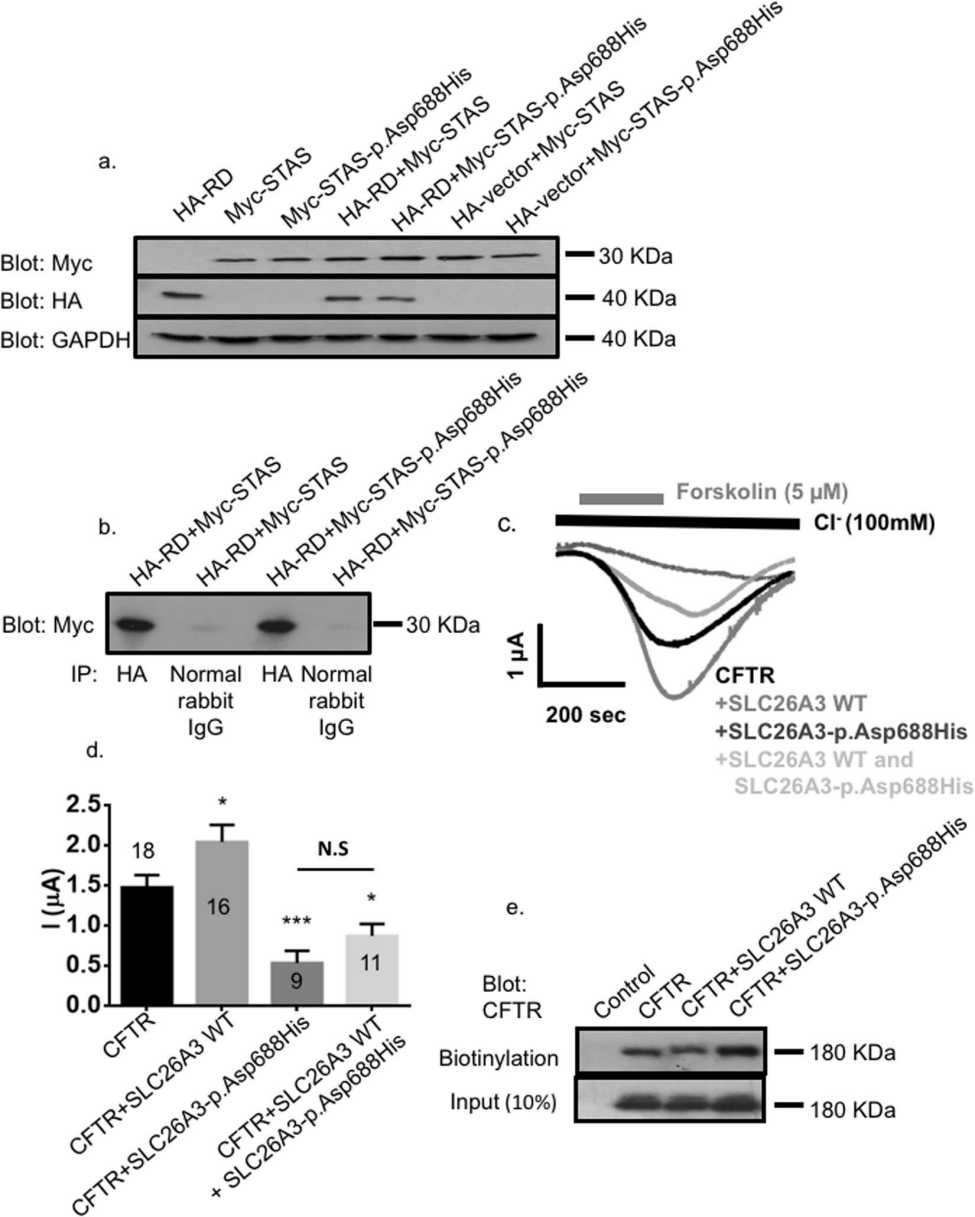
The effects of SLC26A3-p.Asp688His mutation on SLC26A3-CFTR interaction and regulation. COS7 cells were transfected or co-transfected either with the HA-tagged CFTR R domain (HA-RD), with the Myc-tagged wild-type SLC26A3 STAS domain (Myc-STAS), with the Myc-tagged SLC26A3 STAS domain with the c.2062 G > C (p.Asp688His) mutation (Myc-STAS-p.Asp688His), or with an empty HA-vector, as indicated in the corresponding lanes. Cell lysates were used for western blotting (**A**) and immunoprecipitation (**B**). Peptides of correct sizes were detected in the immunoblots by using the anti-Myc, anti-HA, or anti-GAPDH antibodies, as indicated. Immunostaining of GAPDH served as a loading control. In the immunoprecipitation experiments (**B**), cells were co-transfected with CFTR HA-RD and with Myc-STAS or Myc-STAS-p.Asp688His. Anti-HA-antibody or normal rabbit IgG (negative control) were used for the precipitation, and anti-Myc-antibody was used for detection on the blot. Extracts show co-immunoprecipitation of the HA-RD with both the wild-type (Myc-STAS) and the mutant p.Asp688His (Myc-STAS-p.Asp688His) STAS domains. (**C**) Representative traces and (**D**) summary of the current mediated by CFTR-dependent Cl^−^ transport in *Xenopus* oocytes expressing CFTR alone or co-expressing CFTR with wild-type (WT) SLC26A3, SLC26A3-p.Asp688His or both. The numbers in the columns indicate the number of experiments for each condition. (**E**) CFTR trafficking to the plasma membrane monitored by using a biotinylation assay in HEK293 cells expressing CFTR, wild-type SLC26A3, or SLC26A3-p.Asp688His as indicated.

To study the influence of p.Asp688His on the binding of the SLC26A3 STAS and CFTR R domains, we prepared constructs of SLC26A3 STAS in pCMV-Myc vector and CFTR R domain in pCMV-HA vector (Clontech, Palo Alto, CA). The mutation c.2062 G > C (p.Asp688His) was engineered by primer-directed mutagenesis both to the STAS domain construct and to the full-length SLC26A3 construct in pcDNA3.1(+) vector (Clontech). The expression of the CFTR R domain, when co-transfected with the mutant STAS-p.Asp688His, led to a slightly lower yield compared with the co-transfection with the wild-type STAS (Fig. 3a). In co-immunoprecipitation experiments, extracts showed co-immunoprecipitation of the HA-tagged R domain with both the Myc-tagged wild-type STAS and with the mutant STAS-p.Asp688His domain (Fig. 3b).

Although our findings showed that the mutant STAS-p.Asp688His domain can bind to the R domain of CFTR, we further assessed the pathogenicity of p.Asp688His by using eukaryotic expression vectors to characterize the interaction within the SLC26A3/CFTR complex and the effect of SLC26A3-p.Asp688His on CFTR channel activity. To this end, we conducted electrophysiological measurements in *Xenopus* oocytes injected with cRNA of full-length CFTR, either alone or together with the wild-type SLC26A3 or the mutant SLC26A3-p.Asp688His. Our measurements showed that unlike the wild-type SLC26A3, which is a strong activator of CFTR-dependent anion transport, SLC26A3-p.Asp688His mutant suppresses CFTR (Fig. 3c and d). In addition, the measurements indicated a dominant negative effect, as the co-expression of CFTR with SLC26A3-p.Asp688His alone or in the presence of the wild-type SLC26A3 attenuated CFTR activity, while the wild-type SLC26A3 normally activated CFTR (Fig. 3c and d). The monitored ~60% inhibition of CFTR-mediated Cl^−^ transport in the presence of wild-type and mutant SLC26A3-p.Asp688His is in line with heterozygosity (Fig. 3d).

**Figure 4:**
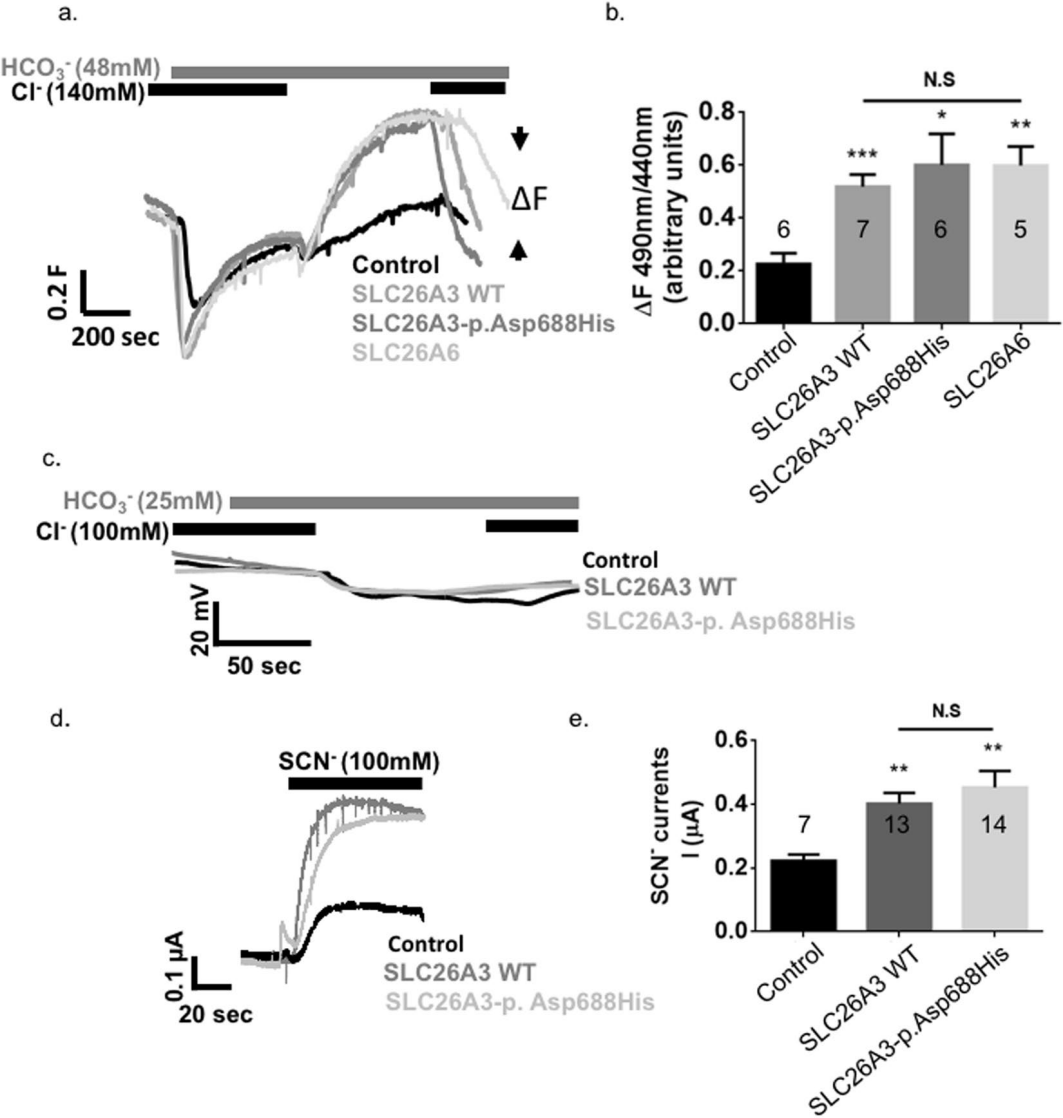
The effects of SLC26A3-p.Asp688His mutation on SLC26A3 function. HEK293 cells were transfected with either empty vector, SLC26A3 wild-type (WT), SLC26A3-p.Asp688His or SLC26A6. Subsequently, pH_i_ changes were monitored as described in the methods section. Both SLC26A3 and SLC26A3-p.Asp688His show similar Cl^−^/HCO_3_^−^ exchange activity as described in (**A**) representative traces and (**B**) summary. (**C**) The Cl^−^/HCO_3_^−^ exchange activity of WT SLC26A3 and SLC26A3-p.Asp688His did not affect the membrane potential of *Xenopus* oocytes suggesting electroneutral activity. However, both WT SLC26A3 and SLC26A3-p.Asp688His generate similar uncoupled SCN^−^ currents as shown in (**D**) representative traces and (**E**) summary.

As the integrity of the STAS domain is required for normal expression and activity of SLC26A3 in mammalian cells^44,45^, we performed functional and expression analyses of SLC26A3 and CFTR. We utilized a biotinylation assay to monitor the effect of the SLC26A3-p.Asp688His mutant on the surface expression of CFTR in mammalian cells. This analysis showed that CFTR trafficking to the plasma membrane is unaffected by the presence of the SLC26A3-p.Asp688His mutant (Fig. 3e). Furthermore, we measured SLC26A3-mediated Cl^−^/HCO_3_^−^ exchange in HEK-293 cells transfected with either wild-type SLC26A3, SLC26A3-p.Asp688His or SLC26A6. Our results indicated that wild-type SLC26A3, SLC26A3-p.Asp688His and SLC26A6 exhibit similar Cl^−^/HCO_3_^−^ exchange activity (Fig. 4a and b). Since changes in transport stoichiometry are expected to affect the electrogenicity of SLC26A3, we monitored the effect of SLC26A3-mediated Cl^−^/HCO_3_^−^ transport on the membrane potential in *Xenopus* oocytes. As shown in Fig. 4c, changes in the membrane potential of *Xenopus* oocytes expressing either wild-type SLC26A3 or SLC26A3-p.Asp688His were similar to control cells, suggesting that both SLC26A3 and SLC26A3-p.Asp688His are electroneutral Cl^−^/HCO_3_^−^ exchangers. This confirms the previously shown electroneutrality of the human SLC26A3 protein with stoichiometry of 1 Cl^−^: 1 HCO_3_^−^^44,46,47^. Finally, to test whether SLC26A3 and SLC26A3-p.Asp688His are functional in *Xenopus* oocytes, we measured SCN^−^ currents, as previously described^48^. As shown in Fig. 4d and e, both proteins mediate similar uncoupled SCN^−^ transport. Taken together, these findings suggest that the SLC26A3-p.Asp688His mutation retains normal Cl^−^/HCO_3_^−^ exchange activity and expression similar to wild-type SLC26A3. Nevertheless, the c.2062 G > C (p.Asp688His) mutation suppresses CFTR, while retaining CFTR trafficking to the plasma membrane.

## Discussion

This study suggests that human OAT and asthenozoospermia are associated with the heterozygous mutation c.2062 G > C (p.Asp688His) in the regulatory STAS domain of SLC26A3. Additionally, we demonstrate that the molecular mechanism behind the male subfertility is likely to be the dominant inhibition of CFTR by the SLC26A3-p.Asp688His mutant. Although sperm counts and morphology of the infertile c.2062 G > C (p.Asp688His) heterozygotes were variable and seminal plasma pH was normal, a striking phenomenon was the small proportion of sperm showing rapid progressive motility, with the median of 10% (WHO class A, normal value >25%) (Fig. 2). Indeed, finding the heterozygous c.2062 G > C (p.Asp688His) allele in 2 controls with unspecified fertility status was followed by observation of the absence of rapid progressive motility of sperm (WHO class A 0%) in their semen samples, providing further evidence for the deleterious role of this mutation.

Since monitoring of the SLC26A3-p.Asp688His mutant showed normal Cl^−^/HCO_3_^−^ exchange activity (Fig. 4 a and b), we focused on the regulatory role of the STAS domain of SLC26A3, which harbors the mutation, in modulation of the CFTR activity. Our results show suppression of CFTR by the SLC26A3-p.Asp688His mutant, yet the interaction between the two proteins is retained (Figs 3 and 4). A similar mechanism with completely different phenotypes was suggested by Ko *et al*., who showed a corresponding inhibition of CFTR by another *SLC26A3* mutation, the mouse homologue for the CLD-associated c.2025_2026insATC (p.Ile675_Arg676insIle)^15^. Similarly to the novel c.2062 G > C (p.Asp688His) reported in the current study, the mouse orthologue for the STAS domain mutant p.Ile675_Arg676insIle interacted with the R domain of CFTR but resulted in impaired CFTR activity^15^. Dirami *et al*. studied the association between the sperm-specific gene *SLC26A8* (alias the testis anion transporter 1, Tat1 [MIM: 608480]) and human asthenozoospermia: in accordance with our functional results, their study revealed that although SLC26A8 and CFTR proteins interacted, the mutated forms of SLC26A8 failed to activate CFTR^22^.

To underline the crucial role of CFTR function in male infertility, (i) defective expression of *CFTR* in human sperm is associated with reduced sperm quality^49^, (ii) homozygosity and heterozygosity for less-pathogenic *CFTR* mutations in humans are associated with reduced fertility without CBAVD^29–36,38^, and iii) the fertilizing capacity of sperm obtained from heterozygous *CFTR* mutant mice is significantly lower than that of wild-type mice^11^. Indeed, milder phenotypes of CF, such as male infertility without other manifestations, are associated with normal Cl^−^ channel activity but with aberrant HCO_3_^−^ secretion only^24,50^, giving support to the crucial role of the interaction between SLC26 transporters and CFTR. A remarkable piece of evidence for heterozygous *SLC26* mutations in modulating CFTR and sperm motility is the association of three unique heterozygous mutations in the STAS domain of *SLC26A8* with human asthenozoospermia^22^.

Unlike the sperm-specific SLC26A8, the loss of which causes complete lack of sperm motility and reduced sperm fertilization potential in mice and asthenozoospermia in humans^22,51,52^, the role of SLC26A3 in regulation of male fertility is more complex. First, SLC26A3 is expressed at multiple sites of the male reproductive tract, from developing spermatocytes and efferent ducts of the testis to the epididymis and seminal vesicle^10,53^. Second, SLC26A3 plays, together with SLC9A3, a major role in the reabsorption of NaCl and fluid in the efferent ducts of the testis, reflected by the CLD-associated low seminal plasma pH and a high Cl^−^ content, in addition to the luminal accumulation of fluid and infertility in both CLD men and *SLC9A3* (MIM: 182307) knockout mice^8,54^. In the current study, the seminal plasma pH in the heterozygotes for the c.2062 G > C (p.Asp688His) mutation was normal, and the Cl^−^/HCO_3_^−^ exchange activity of the SLC26A3-p.Asp688His mutant retained in transfected cells, indicating a normal SLC26A3 anion transport activity along the male reproductive tract epithelia (Figs 2 and 4). Consequently, some of the CLD-related STAS domain mutants are partly active *in vitro*, and even deletion of the entire C-terminal cytoplasmic domain is associated with partial activity of the SLC26A3-mediated anion transport^14,44,45^. Thereby, the sperm motility defect of the c.2062 G > C (p.Asp688His) heterozygotes, together with suppression of CFTR by the SLC26A3-p.Asp688His mutant in overexpression systems, propose a role for impaired SLC26A3/CFTR function in human sperm rather than the male reproductive tract epithelia. Notably, the Cl^−^ concentration in seminal fluid is around 30 to 40 mM^55^. Under these conditions, SLC26A3-mediated HCO_3_^−^ transport is expected to be significantly higher compared with that of CFTR, which is strongly inhibited by extracellular Cl^−^^56,57^.

The SLC26 transporters are associated with recessive diseases: *SLC26A2* (MIM: 606718) with diastrophic dysplasia (MIM: 222600), *SLC26A3* (MIM: 126650) with CLD (MIM: 214700), *SLC26A4* (MIM: 605646) with Pendred syndrome (MIM: 274600), and *SLC26A5* (MIM: 604943) with deafness (MIM: 613865), without evidence of any disease-related features in heterozygous individuals^23^. Correspondingly, men who father a child with CLD and carry the c.949_951delGTG (p.Val318del) mutation possess no phenotypic features of CLD and have no major fertility-related problems (S.W., unpublished data), although data on their semen findings are lacking. Our series with three heterozygotes for the c.949_951delGTG (p.Val318del) mutation (Table 1) revealed severe OAT and, in one of the subjects, azoospermia to which an additional contributor may be the heterozygosity of the *CFTR 5 T* allele. Unfortunately, we had no data on seminal plasma pH of those three men to assess the influence of the heterozygous c.949_951delGTG (p.Val318del) mutation on SLC26A3 activity. Nevertheless, this study suggests that human OAT and asthenozoospermia are associated with the heterozygous mutation c.2062 G > C (p.Asp688His) in the STAS domain of  *SLC26A3*, and most probably, a feature that modulates the fertility in men carrying the loss-of-function allele c.949_951delGTG (p.Val318del) as reported here. The notion that the p.Asp688His variant has never been found in Finnish patients with CLD is consistent with its normal anion exchange activity and impairment of only the SLC26A3/CFTR complex (Figs 3 and 4). The haploid state of the germ cells might make heterozygous alterations deleterious only for sperm motility and fertility, without the intestinal symptoms of CLD. As even none of the p.Val318del heterozygotes had spermatoceles reported in CLD^8^, heterozygous mutations of *SLC26A3* along the male reproductive tract epithelia are likely to be tolerated, or compensated by other SLC26 transporters, as shown for SLC26A6 upregulation in the SLC26A3-defective intestine^9^.

The observed 1.1% heterozygosity the Finnish founder mutation for CLD, c.949_951delGTG (p.Val318del), is comparable to that of the general population where the incidence of homozygosity and CLD is around 1:30,000–1:40,000^22^. The prevalence of c.2062 G > C (p.Asp688His) mutation, with the heterozygosity of 3.2% in infertile men and 1.4% in the Finnish population, is higher than that of the founder mutation and has the estimated homozygosity of 1:20,000. These data suggest that c.2062 G > C (p.Asp688His) is, indeed, a founder mutation enriched in Finland for the unique population history with small original subpopulations, local founder effects, and genetic bottlenecks^41,58^. Unfortunately, we were unable to obtain samples from the relatives of the c.2062 G > C (p.Asp688His) heterozygotes to study maternal effects. Despite the fact that homozygous mutations remain non-existent both in this study and in the 106 heterozygotes previously reported (ExAC), c.2062 G > C (p.Asp688His) is likely to be inherited through the affected sperm, similar to the founder mutation for CLD. Also supporting paternal segregation is the relatively high prevalence of this mutation in the Finnish population. The fact that even CLD-related sperm findings for sperm motility and seminal plasma pH vary (Fig. 2) highlights the clinical phenotype of subfertility—rather than complete infertility—in relation to *SLC26A3* mutations.

Beyond sperm motility, compromised function of SLC26A3 is likely to modulate sperm capacitation, hyperactivation, and acrosome reaction, the processes in which SLC26A3 mediates HCO_3_^−^ entry into spermatozoa by working in parallel with CFTR^12,13,59–62^. Indeed, HCO_3_^−^ related capacitation events can be inhibited by the removal of Cl^−^, by CFTR inhibitor, or by SLC26A3 inhibitor and antibody in rodents^12^. Further studies on members of the SLC26 family are likely to provide important data on the pathogenesis of male infertility and the action of the SLC26A3/CFTR complex in sperm cells.

## Methods

Our study comprised 283 Finnish men with idiopathic infertility who had been recruited during fertility assessment at the university centers for reproductive medicine. The men had abnormal sperm according to the WHO criteria^63^. 91 (32%) of 283 men in our sample had a diagnosis of oligoasthenoteratozoospermia (OAT), 79 (28%) severe OAT, 32 (11%) azoospermia, 40 (14%) oligoteratozoospermia, 19 (7%) teratozoospermia, 7 (2%) oligoastheno-/asthenozoospermia, and 15 (5%) asthenoteratozoospermia. Chromosomal and hormonal abnormalities and Y chromosomal microdeletions had been excluded. The control group of Finnish men (n = 211) comprised 51 healthy men with proved fertility, 132 healthy men with an unknown fertility status, and 28 men with an established cause of infertility, such as Y chromosomal deletion, Klinefelter syndrome, chromosomal translocation, or obstruction. After standard DNA extraction from venous blood samples, we analyzed the 21 exons and exon/intron boundaries of *SLC26A3* by direct sequencing with intronic primers^64^. We also tested the frequency of the *5 T* allele of *CFTR* by sequencing with specific primers^65^, and searched for *CFTR* mutations among the heterozygotes of *SLC26A3* variants or of the *5T* allele by using INNO-LiPA *CFTR*17 + Tn and *CFTR*19 test strips (Innogenetics, Gent, Belgium), recognizing ≈87% of Finnish *CFTR* mutations^66^. All methods were performed in accordance with the relevant guidelines and regulations. The human study protocol was approved by the Institutional Review Boards at Helsinki, Turku, and Oulu University Hospitals, and informed consent was obtained from all participants.

### Co-immunoprecipitation studies of the R domain of CFTR and STAS domain of SLC26A3

We prepared constructs of SLC26A3 STAS (amino acids 518-713: N-terminal residue NIYKNKKDY and C-terminal residue KSSIFFLTI) in pCMV-Myc vector (N-terminal Myc-tag) and CFTR R domain (amino acids 590-858; N-terminal residue CVCKLMANK and C-terminal residue LRYITVHKS) in pCMV-HA (N-terminal HA tag) vector (Clontech, Palo Alto, CA)^15^, and used primer-directed mutagenesis to engineer the mutation c.2062 G > C (p.Asp688His) both to the STAS domain construct and, for the electrophysiological measurements, to the full-length SLC26A3 construct (N-terminal residue MIEPFGNQY) in pcDNA3.1(+) vector (Clontech), excised from the original pCMV5 vector^5^. All constructs were verified by sequencing and their expression was confirmed in COS7 cells.

The experimental procedure included normalization of the DNA amounts by using the empty vector in each transfection. Precipitation was achieved according to the instructions of the Immunoprecipitation Kit with protein G-agarose (Roche, Basel, Switzerland). Pierce^TM^ BCA Protein Assay Kit (Thermo Fisher Scientific, Waltham, MA) was used to assess the amount of proteins, and immunoblotting was performed with the anti-HA (1:500) and anti-Myc (1:100) antibodies (Clontech). Staining with GAPDH antibody (Santa Cruz Biotechnology, Dallas, TX) served as an endogenous control. In co-immunoprecipitation experiments, cell lysates were incubated with the anti-HA antibody (3–5 µg/mL) or with a normal rabbit IgG (control), followed by precipitation with protein-G agarose, separation of precipitated peptides by SDS-PAGE, and immunoblotting with the anti-Myc antibody (1:100) (Clontech). Horseradish peroxidase-conjugated antibodies (Sigma-Aldrich, St. Louis, MO) were used as secondary antibodies and signals were detected with an enhanced-chemiluminescence (ECL) substrate (GE Healthcare, Chicago, IL).

### Electrophysiological measurements and surface expression

The CFTR and SLC26A3 constructs in a pcDNA3.1(+) vector (Clontech) were linearized using restriction enzymes. *In vitro* transcription was then performed using the AmpliCap-Max™ T7 High Yield Message Maker Kit (CELLSCRIPT, Madison, WI).

Oocytes were isolated from *Xenopus laevis*. All experiments and methods were performed in accordance with relevant guidelines and regulations, and the protocol was approved by the Ben Gurion University of the Negev animal care and use committee. Oocytes were injected with cRNA as indicated (CFTR 16ng, SLC26A3 8ng, p.Asp688His 8ng or equal volumes of water to a total of 32 nl per oocyte). Subsequently, the oocytes were incubated at 18 °C, and tested 48–72 h after cRNA injection. Current recordings were performed with a two-electrode voltage clamp as described elsewhere^25,48^. Briefly, the transport was initiated by adding 5 µM forskolin to the perfusate in the presence of Cl^−^, as indicated. CFTR-dependent currents were recorded at a holding membrane potential of −60 mV. Data were analyzed by using the Clampex 10.5 system (Axon Instruments Inc., Union City, CA). The electrodes were backfilled with a 3 M KCl solution. The following solution was used as indicated in the figures (100 mM Cl^−^): standard HEPES-buffered ND96 oocyte regular medium containing (in mM) 96 NaCl, 2 KCl, 1.8 CaCl2, 1 MgCl2, and 5 HEPES, pH 7.5, with or without forskolin (5 µM). HCO_3_^−^-buffered solutions were prepared by replacing 25 mM Na^+^-anion with 25 mM Na^+^-HCO_3_^−^. HCO_3_^−^-buffered solutions were gassed with 5% CO_2_ and 95% O_2_. Solutions containing SCN^−^ were prepared by the replacement of NaCl and KCl with the respective SCN^−^ salts. Results are shown as means + SEM with the statistical significance set at P < 0.05 and calculated with Student’s t-test. To monitor CFTR trafficking, we used a modified biotinylation assay as previously described^48^. Briefly, transfected cells were washed and incubated with EZ-Link Sulfo-NHS-SS-Biotin (0.5 mg/ml; Thermo Fisher Scientific) for 30 min on ice. The biotin was quenched with 50 mM glycine and lysates were prepared with a lysis buffer containing 1 × PBS, 1 mM NaVO3, 10 mM Na-pyrophosphate, 50 mM NaF, pH 7.4, and 1% Triton X-100. Finally, 100 μl of a 1:1 slurry of immobilized neutravidin beads (Thermo Fisher Scientific) were added to 0.5 mL of cell extracts and incubated overnight. Beads were washed with a binding buffer, and proteins were released with 50 μL of SDS-loading buffer. Extracts were loaded onto 8% Tris-glycine SDS-PAGE gels, which were subsequently transferred onto a PVDF membrane and probed with anti-CFTR monoclonal antibody (Merck Millipore, Darmstadt, Germany) diluted at 1:600. Membranes were then incubated with a secondary goat anti–mouse IgG peroxidase conjugate antibody (Thermo Fisher Scientific), diluted at 1:1,000.

### Measurement of intracellular pH

HEK293 cells were transfected with slc26a3 and SLC26A3-p.Asp688His in pcDNA3.1(+) or SLC26A6 in PCMV6-AC-mKate (C-terminal mKate tag – Origene, MD). Cells were attached to coverslips and pH_i_ was measured using an imaging system that consisted of an Eclipse Ti inverted microscope (Nikon, Japan), PE-4000 LED monochromator (CoolLEd, UK) and Hamamatsu flash 4.0LT camera (Hamamatsu photonics, Japan). Fluorescent images were acquired and analyzed with NIS elements software (Nikon, Japan). Cells were loaded on stage with 2 µM BCECF-AM and perfused with the indicated solutions. Regular solution (containing in mM: 140 NaCl, 5 KCl, 1 MgCl2, 10 HEPES, 1 CaCl2, 10 glucose, pH adjusted to 7.4). Cl^−^ free solutions were prepared by the replacement of Cl^−^ with gluconate. HCO_3_^−^-buffered solutions were prepared by replacing 25 mM or 48 mM Na^+^-anion with 25 mM or 48 mM Na^+^-HCO_3_^−^ and reducing HEPES to 5 mM. HCO_3_^−^-buffered solutions were gassed with 5% CO_2_ and 95% O_2_.

### Data availability

All data generated or analysed during this study are included in this published article.

## Electronic supplementary material

Original gels and blots
