# Supplementary material for: A missense mutation in SLC26A3 is associated with human male subfertility and impaired activation of CFTR
Source: Sci Rep. 2017 Oct 27;7:14208. doi: 10.1038/s41598-017-14606-3 (PMC5660164; doi:10.1038/s41598-017-14606-3)
Supplement: Supplementary file 1 — Original gels and blots [file 41598_2017_14606_MOESM1_ESM.pdf]

A missense mutation in *SLC26A3* is associated with human male subfertility and impaired activation of CFTR

Satu Wedenoja, MD, PhD,[1]* Ahlam Khamaysi,[2] Liana Shimshilashvili,[2] Shireen Anbtawe-Jomaa,[2] Outi Elomaa, PhD,[3] Jorma Toppari, MD, PhD,[4] Pia Höglund, MD, PhD,[5] Kristiina Aittomäki, MD, PhD,[6] Christer Holmberg, MD, PhD,[7] Outi Hovatta, MD, PhD,[8] Juha S. Tapanainen, MD, PhD,[1,9] Ehud Ohana, PhD,[2]**♦ and Juha Kere, MD, PhD[3,10,11]♦

♦These authors contributed equally to this work.

[1]Obstetrics and Gynecology, University of Helsinki and Helsinki University Hospital, FI-00014 Helsinki, Finland
[2]Department of Clinical Biochemistry and Pharmacology, Faculty of Health Sciences, Ben-Gurion University of the Negev, Beer-Sheva, Israel
[3]Folkhälsan Institute of Genetics, and Molecular Neurology Research Program, University of Helsinki, FI-00014 Helsinki, Finland
[4]Department of Physiology, Institute of Biomedicine, University of Turku, and Department of Pediatrics, Turku University Hospital, FI-20014 Turku, Finland
[5]City of Kauniainen, Health Care Services, FI-02700 Kauniainen, Finland
[6]HUSLAB, Laboratory of Genetics, Helsinki University Hospital, and Genome-Scale Biology research program, University of Helsinki, FI-00029 Helsinki, Finland
[7]Hospital for Children and Adolescents, University of Helsinki and Helsinki University Hospital, FI-00014 Helsinki, Finland
[8]Department of Clinical Science, Karolinska Institutet, SE-17177 Stockholm, Sweden
[9]Obstetrics and Gynecology, University of Oulu and Oulu University Hospital, FI-90220 Oulu, Finland
[10]Department of Biosciences and Nutrition, Karolinska Institutet, SE-14183 Huddinge, Sweden
[11]Department of Medical & Molecular Genetics, King's College London, London SE1 9RT, England

Correspondence to: *satu.wedenoja@helsinki.fi and **ohanaeh@bgu.ac.il

Figure 3a.

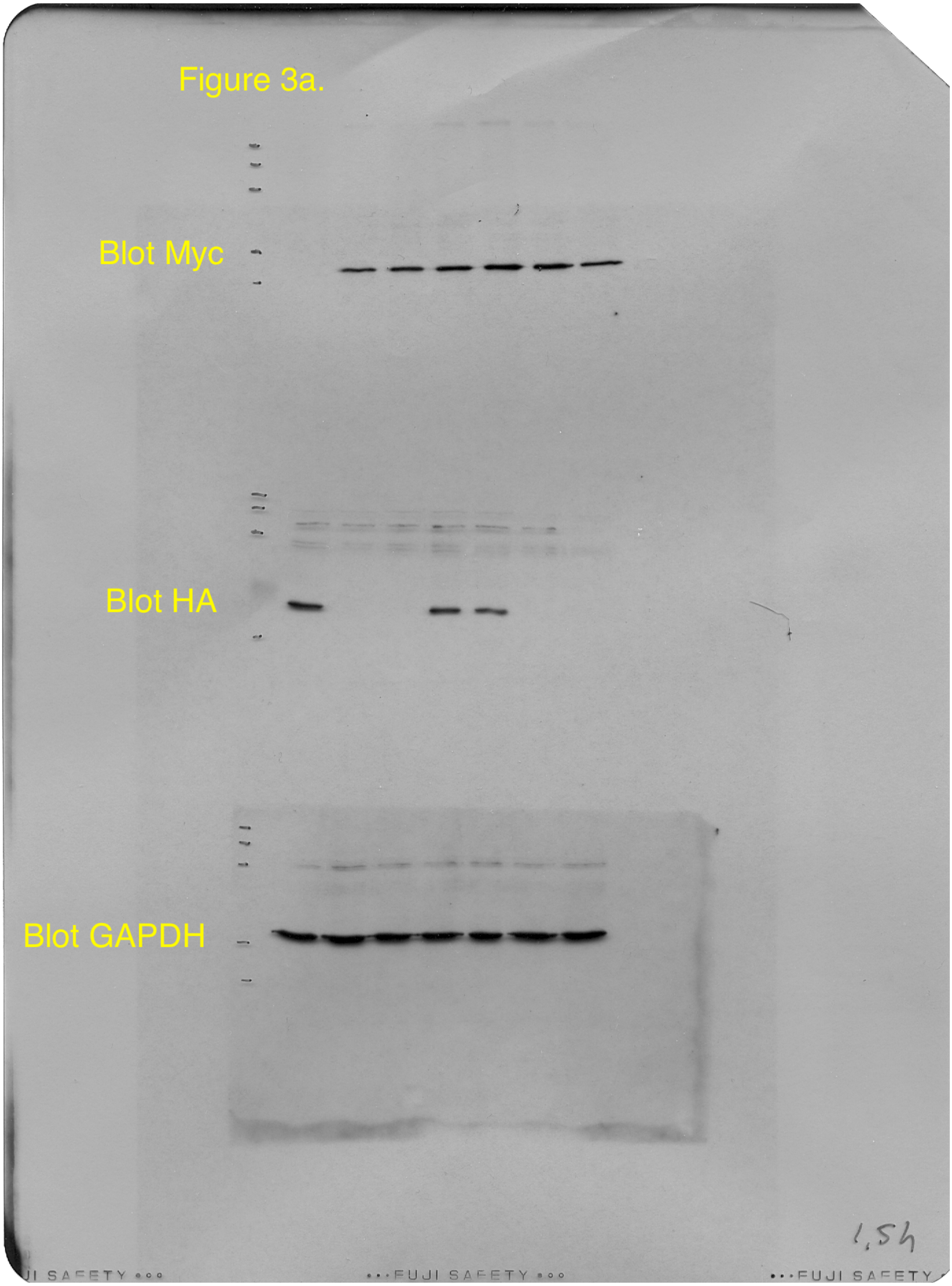

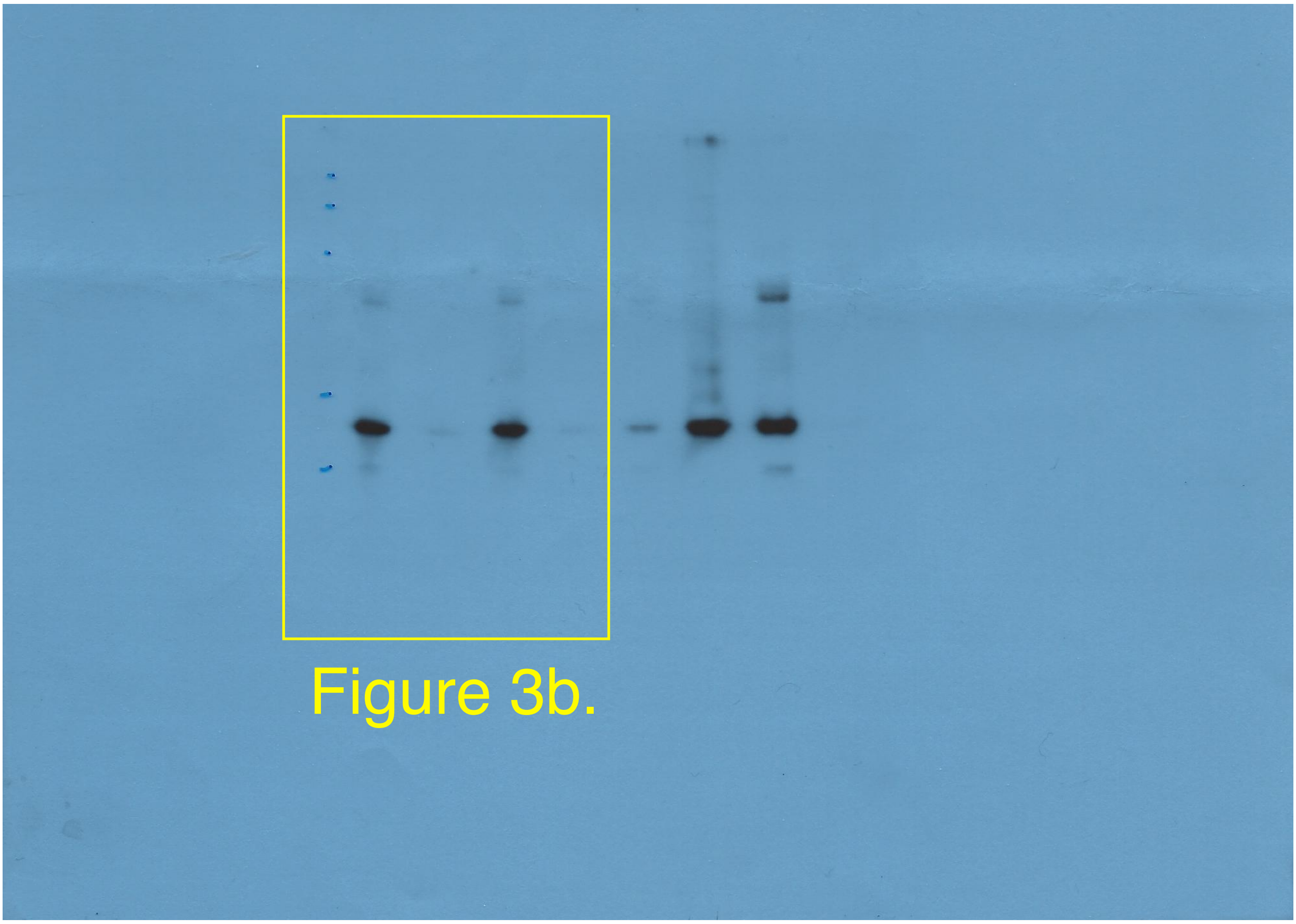

Figure 3b.

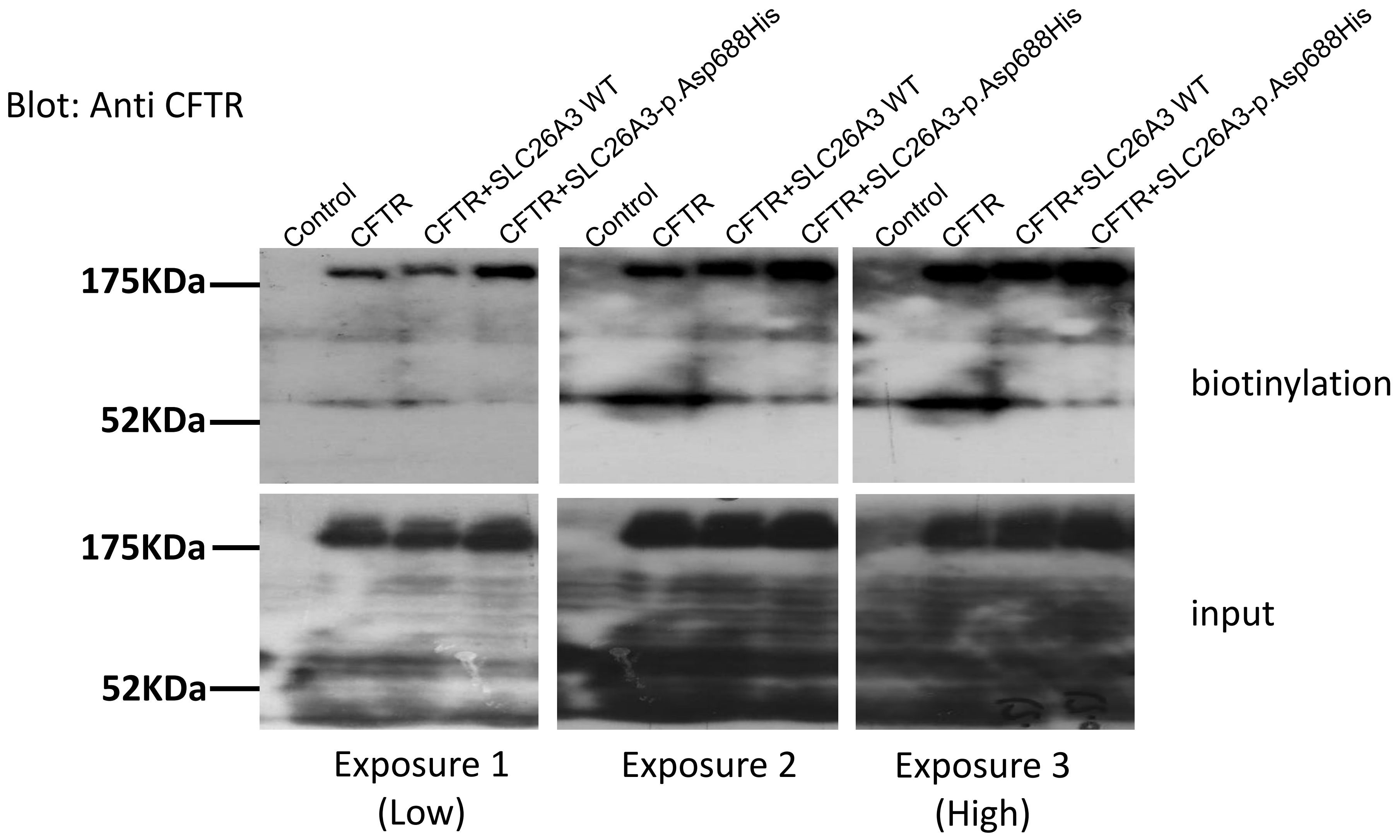
Blot: Anti CFTR
Control
CFTR
CFTR+SLC26A3 WT
CFTR+SLC26A3-p.Asp688His
Control
CFTR
CFTR+SLC26A3 WT
CFTR+SLC26A3-p.Asp688His
Control
CFTR
CFTR+SLC26A3 WT
CFTR+SLC26A3-p.Asp688His
175KDa
52KDa
175KDa
52KDa
biotinylation
input
Exposure 1
(Low)
Exposure 2
Exposure 3
(High)
